# Emissions and leachate profile of MSW disposal sites of metropolitan cities of Pakistan using LandGEM model

**DOI:** 10.1186/s13021-026-00403-x

**Published:** 2026-02-03

**Authors:** Bibi Ilmas, Sofia Khalid, M. Ijaz, Imtiaz Hussain

**Affiliations:** 1 Department of Environmental Sciences, Allama Iqbal Open University, H-8 Campus, Islamabad, 44000 Pakistan; 2 Global Change Impact Studies Centre (GCISC), Ministry of Climate Change, Government of Pakistan, Islamabad, Pakistan; 3 Pakistan Environmental Protection Agency, Ministry of Climate Change & Coordination, Government of Pakistan, Islamabad, Pakistan

**Keywords:** Municipal solid waste, Methane emissions, Leachate generation, Environmental monitoring, Landfill gas capture, Climate change mitigation

## Abstract

The rapid increase in municipal solid waste (MSW) generation across urban centers in Pakistan, combined with insufficient waste management infrastructure, presents a significant environmental and public health challenge. This study assesses methane emissions and leachate generation from major MSW dumpsites in Rawalpindi and Lahore, two of Punjab province’s largest cities. Emissions were estimated and projected over a 50-year active timespan using the U.S. EPA LandGEM model following IPCC 2006 guidelines. Cumulative emissions from Lahore’s solid waste disposal (SWD) systems were calculated at approximately 133,446 Gg, equivalent to 108 Mt CO₂-eq, with contributions comprising 26% methane, 73% carbon dioxide (CO₂), and 0.2% non-methane organic compounds (NMOCs). In contrast, Rawalpindi’s SWD systems generated 958 Gg (or 7.8 Mt CO₂-eq) over their operational life, exhibiting a similar emissions profile. Two unmanaged Lahore sites—LD2 (1643 Gg CH₄) and MB1 (1383.9 Gg CH₄)—emerged as the most significant methane emitters across both cities. These results underscore the urgent need for targeted waste management strategies, particularly the deployment of methane capture technologies and effective leachate treatment systems. The study highlights the substantial greenhouse gas emissions and groundwater contamination risks posed by unmanaged landfills. To mitigate these impacts and align with national climate goals, the adoption of site-specific policies and sustainable waste-to-energy solutions is imperative.

## Introduction

Global warming and climate change, primarily driven by the accumulation of greenhouse gases (GHGs) such as carbon dioxide (CO₂) and methane (CH₄), remain among the most urgent environmental crises of our time. These gases disrupt ecological stability, impact human health, and intensify the frequency and severity of extreme weather events worldwide [46]. The waste sector is a notable contributor to global greenhouse gas (GHG) emissions. Landfill gases (LFGs) are a significant by-product of the anaerobic decomposition of organic components in municipal solid waste (MSW), along with hazardous leachate in landfills and open dumpsites [43, 50].

LFGs are primarily composed of methane (CH₄) and carbon dioxide (CO₂), along with trace amounts of non-methane organic compounds (NMOCs) and volatile organic compounds (VOCs) (IPCC [63]). Methane, a potent greenhouse gas with a global warming potential 25 times that of CO₂ over a 100-year timeframe, is particularly concerning due to its role in accelerating climate change [16]. The generation of landfill gas typically begins within months of waste deposition and can continue for decades, depending on the composition, moisture content, temperature, and management practices of the landfill (U.S. [32]).In developing countries, including Pakistan, the impact is more pronounced because of inadequate waste collection, lack of segregation at source, and the prevalence of unmanaged disposal methods, which result in higher rates of uncontrolled methane emissions [21, 60].

Leachate composition and volume are affected by various factors, including waste characteristics, moisture levels, climatic conditions, and the compaction and degradation processes in dumpsites [44]. In South Asian countries like Pakistan, poorly managed dumpsites often lead to leachate accumulation without appropriate containment or treatment, threatening soil, surface water, and groundwater quality [13]. The lack of bottom liners, leachate collection systems, and impermeable covers facilitates contaminant migration into adjacent ecosystems, resulting in long-term environmental degradation and public health risks [12, 48, 64]. Implementing engineered landfill designs, leachate collection and treatment systems, and systematic site monitoring is crucial to mitigate these environmental impacts [66].

The quantity and rate of LFG production are commonly estimated using models such as the IPCC’s First Order Decay (FOD) method or the U.S. EPA’s LandGEM model, which require parameters like the methane generation potential (Lo) and methane generation rate constant (k) (IPCC [63]; U.S. [32]). Effective quantification and management of landfill gases are essential not only for climate change mitigation but also for capturing methane as a renewable energy source, thereby aligning waste management with circular economy principles [31].

In Pakistan, MSW management is characterized by systemic challenges, including a lack of segregation at source, informal waste collection, limited recycling infrastructure, and reliance on open dumping or unmanaged landfills. These issues not only pose significant public health risks but also result in elevated and poorly documented methane emissions. In Pakistan, the waste sector contributes about 3% to the national GHG inventory, with landfill methane emissions accounting for nearly 73% of the sector’s total emissions [36]. These emissions are expected to increase significantly due to rapid urbanization, projected to grow at an annual rate of 3.65% (ADB [62]), and rising per capita waste generation. Despite its relatively small share of total emissions, the waste sector presents a substantial opportunity for low-cost mitigation through improved landfill management, composting, recycling, and methane capture technologies [65]. Studies such as those by [14], Zuberi et al. [61], and Korai et al. [29] have attempted to model GHG emissions using basic first-order decay (FOD) models or tools like the U.S. EPA’s LandGEM. However, these efforts have largely been limited to specific cities with inconsistent input parameters and a lack of integration with Pakistan-specific waste characteristics. Furthermore, Pakistan continues to rely on Tier 1 methodologies under the 2006 IPCC Guidelines, using default emission factors and without incorporating localized waste composition, climatic conditions, or landfill management practices. This results in substantial uncertainties in national GHG inventories. In contrast, countries with advanced waste systems have transitioned to Tier 2 or Tier 3 approaches with site-specific emission factors and robust data collection frameworks, leading to more accurate estimations and targeted mitigation strategies.

Despite these findings, a critical research gap exists in the form of updated, region-specific emission baselines for solid waste disposal sites using locally calibrated models. Current estimates are not only fragmented but also inadequate for informing policy-level decisions aligned with Pakistan’s Nationally Determined Contributions (NDCs) under the Paris Agreement.

Therefore, this study aims to develop a robust, localized baseline of methane emissions from selected MSW disposal sites in Pakistan using improved modeling approaches and accurate field-based parameters. This will provide a more realistic representation of the sector’s contribution to national GHG inventories and support evidence-based mitigation policies.

### Present study

**Fig. 1:**
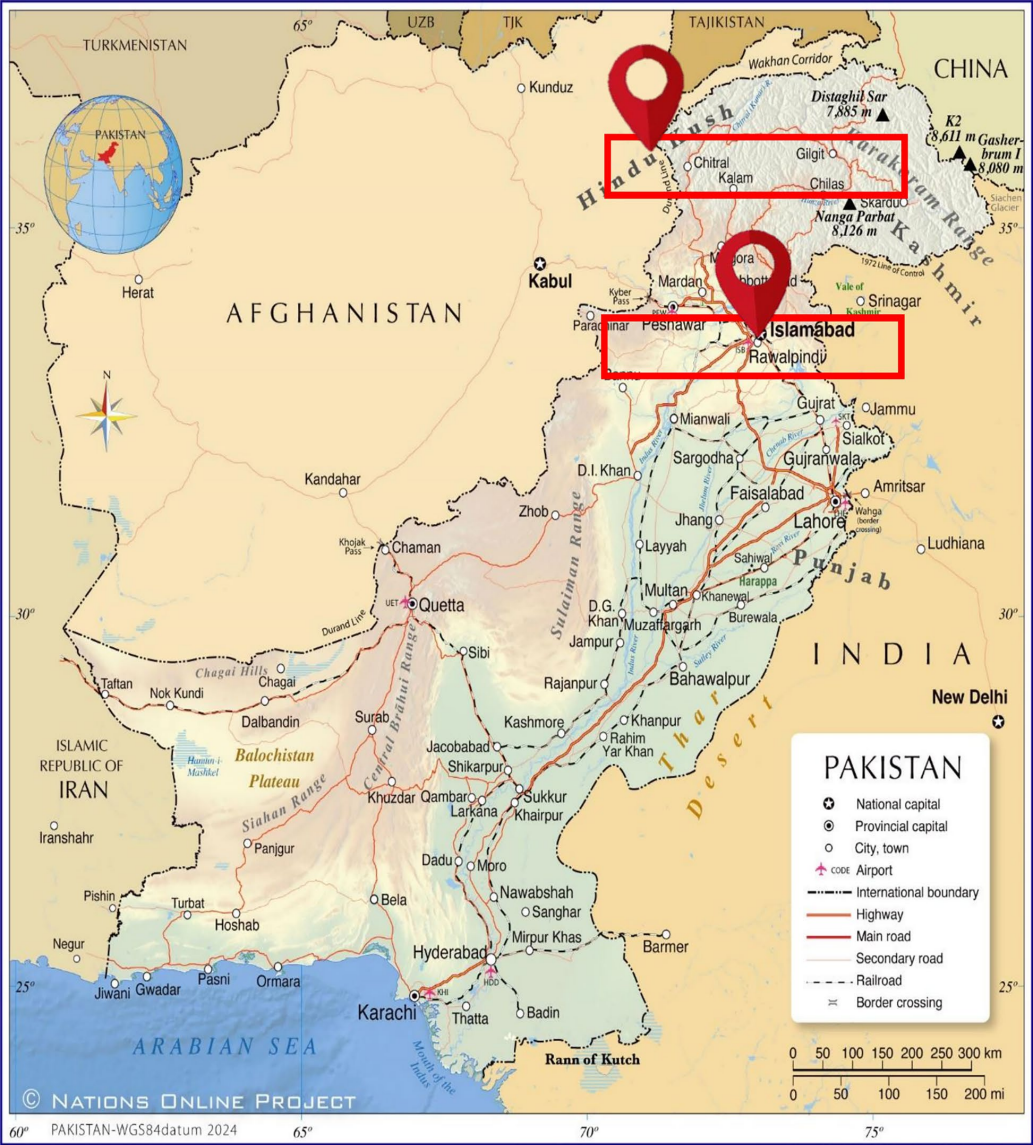
HYPERLINK "sps:id::fig1||locator::gr1||MediaObject::0" Pakistan map showing studied sites of Rawalpindi & Lahore

The objective of this study is to quantify greenhouse gas (GHG) emissions and leachate estimations from municipal solid waste (MSW) disposal sites in two major cities of Punjab, Pakistan: Rawalpindi and Lahore (Fig 1). Utilizing the LandGem mathematical model version 3.02, this research endeavours to provide a comprehensive emissions profile for these metropolitan cities and to propose strategies for mitigating these emissions. Punjab is Pakistan’s most populous province, with an estimated population of 110 million and a growth rate of 2.17% (Pakistan Bureau of Statistics [40]).

The emissions data gathered from the studied cities can serve as a valuable benchmark for evaluating waste sector emissions in other regions of Pakistan. This assessment included on-site evaluations of dumpsites and aimed to inform policy development while enhancing sustainable waste management practices. Ultimately, this research seeks to bolster Pakistan’s efforts to combat climate change and provide critical data for local governments and environmental agencies to mitigate the environmental impact of urban waste and strengthen the resilience of cities.

## Materials and methods

### Present solid waste management infrastructure

An assessment of the existing infrastructure for municipal solid waste management has been carried out through visits to various relevant departments. These include the Punjab Environmental Protection Agency (EPA) offices in Lahore and Rawalpindi, the Cantonment boards (CBs) of both cities, such as Walton Cantonment Board, Lahore Cantonment Board (LCB) of Lahore, and CCB (Chaklala Cantonment Board), RCB (Rawalpindi Cantonment Board) of Rawalpindi, the Rawalpindi Waste Management Company (RWMC), and the Lahore Waste Management Company (LWMC).

#### Rawalpindi situation

Rawalpindi generates approximately 0.61 to 0.28 kilograms of municipal solid waste per capita daily, totaling an estimated 1200 to 1300 tons [4, 24, 27]. Approximately 70 to 80 percent of this waste is collected, while the remainder is managed by informal sectors, incinerated, or left uncollected. Open dumping constitutes over 80 percent of the waste management strategy. The Rawalpindi Waste Management Company (RWMC), established in 2014, oversees waste collection and disposal at the Losar Landfill via a transfer station on Murree Road, but does not include waste recovery or treatment processes. The Rawalpindi Cantonment Board (RCB) and Chaklala Cantonment Board (CCB) manage waste within their jurisdictions, disposing of it at temporary dumpsites located at a distance from the city center. These sites are leased for defined periods and subsequently levelled and repurposed for construction. This practice poses significant risks to residents, including the potential for methane-induced explosions and fires, with both cantonments currently utilizing rented land at the ‘Chakri’ site for disposal activities.

#### Lahore situation

Lahore generates approximately 6,000–6500 tons of waste daily, with a per capita rate averaging 0.61 kilograms per day [20]. The city is divided into nine towns, with Ravi Town as the largest contributor at 607 tons per day, followed by Samanabad and Allama Iqbal Towns at 593 and 591 tons, respectively. The Lahore Waste Management Company (LWMC) manages waste disposal at the Lakhodair Sanitary Landfill (27% of municipal solid waste) and the Lakhodair dumping site (73%). The decommissioned Mahmood Booti site still poses challenges, such as odors and environmental contamination. Around 25% of residential societies in Lahore are managed by housing administrations serviced by the LWMC. Informal recycling by sanitation workers is common, with mixed waste often disposed of openly. “Lahore Compost” is estimated to recycle 700 to 1,000 tons of waste daily, along with an unknown amount being recycled illicitly by scavengers who may burn waste to recover materials, contributing to environmental pollution.

### Methodology for emissions profiling

The emissions from various disposal sites in the study areas were analysed using the First Order Decay (FOD) method preferred by the Intergovernmental Panel on Climate Change (IPCC). LandGEM Model version 3.1(January 2025) was used, which is based on a first-order decomposition rate equation (Eq. 1) for quantifying emissions from the decomposition of landfilled waste in municipal solid waste (MSW) landfills.

1 $$\begin{aligned}{G}_{CH4}= & {\sum }_{x=S}^{T-1}{W}_{x}\times MCF\times DOC\times \\& {DOC}_{f}\times F\times \frac{16}{12}\times \left({e}^{-k(T-x-1)}-{e}^{-k(T-x)}\right) \end{aligned}$$

whereas

G_CH4_ = Modeled methane generation rate in reporting year T (metric tons CH_4_)

x = Year in which waste was disposed

W_x_ = Quantity of waste disposed in the landfill in year x (metric tons)

DOC = Degradable organic carbon (Mg C/Mg waste)

T = Reporting year for which emissions are calculated

S = Start year of calculation

MCF = Methane correction factor (fraction)

DOC_f_ = Fraction of DOC dissimilated (fraction)

F = Fraction by volume of CH4 in landfill gas

k = Rate constant (yr^−1^)

*Model Input Parameters* are established in Table 1.

**Table 1:** Model input parameters

Parameter	Unit	Input value	Reference/justification
k = Rate constant year^−1^	Fraction	0.09	1. Warm, arid to semi-arid climatic conditions, High biodegradability, and poor landfill practices [60] 2. Similar regional studies (India, Bangladesh, Indonesia): Often use *k* = 0.06 to 0.1 yr⁻^1^ for open dumpsites with high food waste content [7, 45]
Potential Methane Generation Capacity, L_o_	m^3^/Mg	70	Ahmed et al. ([3]2021) (site-component conservative)
NMOC Concentration	ppmv as hexane	600	U.S. EPA LandGEM Default
Wx = Quantity of waste disposed in the landfill in year x	(Mg)	346,750 Mg/yr (Rawalpindi) 2,372,500 Mg/yr (Lahore)	Site waste records from relevant authorities
DOC = Degradable organic carbon	(g C/g waste)	Inventory Bulk waste—0.17	Pakistan-specific studies (e.g., UNEP [68], Pakistan [22, 32, 49])
MCF = Methane correction factor	(fraction)	0.8 Unmanaged, deep > 5 m unmanaged shallow site	IPCC ([63], 2019) defaults; Pakistan landfill studies [22, 49]
DOC_f_ = Fraction of DOC dissimilated	(fraction)	0.5	[22, 49]
F = Fraction by volume of CH_4_ in landfill gas	(fraction)	0.5	1. U.S. EPA LandGEM [32] – default CH₄ content: 50%(0.5) 2. Zuberi & Ali [60] – modelled 50% CH₄ content in Pakistan dumpsites 3. Batool & Nawaz [13] – CH₄ content estimated ~ 47–50% in Lahore MSW

### Leachate modelling and sensitivity analysis

Leachate generation is calculated for a recent year 2024, using the method adopted by [23] and [37].

2 $$V \, = \, 0.15 \, \times \, R \, \times \, A$$

where

V = volume of leachate discharge in a year (m3/year)

R = annual rainfall (mm)

A = surface area of the landfill (m2)

R and A values are site-specific. Annual rainfall was taken from the Pakistan Meteorological Department (PMD). The method employs a coefficient of 0.15 to encompass all landfill losses without considering multiple parameters. The total leachate quantity is typically more than 75% of precipitation during active and closed phases. The peak leachate rate is influenced by peak precipitation and waste height. If peak precipitation aligns with minimal waste height, the peak leachate rate is directly proportional to the precipitation rate. However, if peak precipitation occurs when the waste height is substantial, there is a delayed effect, resulting in a peak leachate rate lower than the peak precipitation rate.

#### Sensitivity analysis

To evaluate the influence of operational factors on leachate generation, a scenario-based sensitivity analysis was performed. The analysis considered two parameters—seasonal waste cover (fraction of rainfall prevented from infiltration) and waste compaction (reduction of percolation due to density increase).

The baseline leachate volumes were derived from monthly rainfall and measured leachate data for each dumpsite. The model was modified under combinations of four cover effectiveness levels (0%, 25%, 50%, 75%) and four compaction factors (1.00, 0.85, 0.70, 0.50) using the expression:

3 $${\mathrm{Q}}_{\mathrm{L},\mathrm{scenario}}={\mathrm{Q}}_{\mathrm{L},\mathrm{baseline}}\times (1-{\mathrm{E}}_{\mathrm{cover}})\times {\mathrm{F}}_{\mathrm{compaction}}$$

This approach, though simplified, enables quantitative comparison of how management practices (covering and compaction) affect annual leachate generation.

## Results and discussions

### Potential emissions sites

**Fig. 2:**
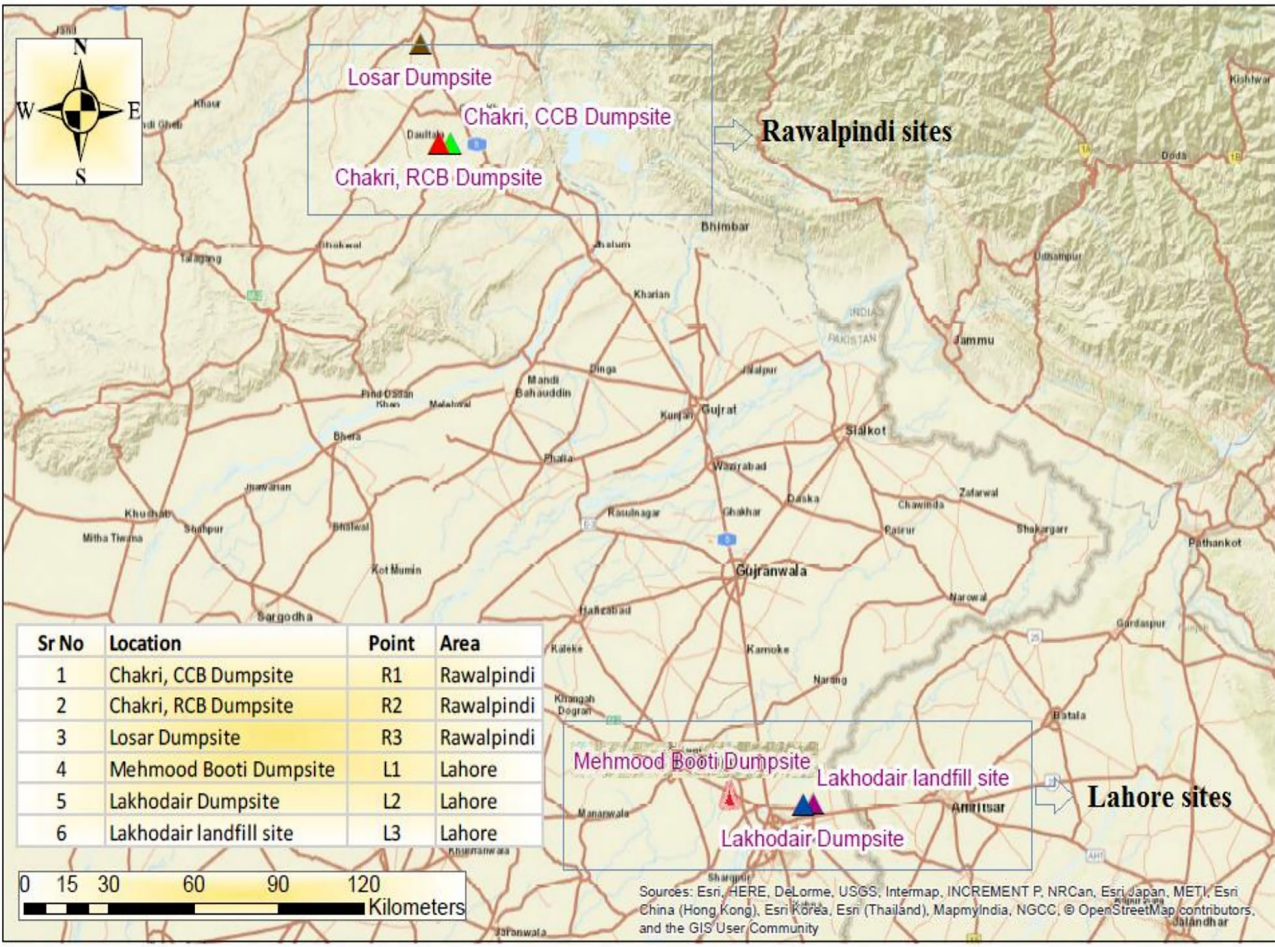
Studied dumpsite location of Rawalpindi & Lahore

This study assesses three municipal final solid waste disposal sites of Lahore and one of Rawalpindi (Figs. 2). Detailed information about these sites is provided in Table 2.

**Table 2:** Attributes of identified solid waste disposal sites

IDs	Rawalpindi	Lahore		
	LD1	MB1	LD2	LF3
SWDs	Losar dumpsite	Mehmood booti Dumpsite	Lakhodair dumpsite	Lakhodair sanitary landfill (Lot I and II)
Location	33°25′13.4"N, 73°11′01.2"E	31˚36′39.90" N 74˚23′08.57" E	31˚37′36.62" N 74˚25′07.64" E	31˚37′36.62" N 74˚25′07.64" E
Started	2003	1997	2016	April, 2016
Closure/Expected closure	2025	2016	2030	2027
Managing authority	RWMC	CDGL	LWMC	LWMC
Disposal site area	30 hectares (300,000 m^2^)	16 hectares (160,000 m^2^)	53 hectares (530,000 m^2^)	20 hectares (200,000 m^2^)
Waste capacity	Not determined	25,00,000 t	Not determined	1,500,000 t
Daily receival (TPD)	900–1000	4000(closed now)	4000	2500
Socioeconomic status of service areas	Urban + rural	Urban + rural areas	Urban	Urban
Waste on-site management	Spreading and compaction and soil layering	Dumping	Spreading and compaction and soil layering	Waste burial in landfill cells
LFG collection system	Nil	Nil	Nil	Gas vents are installed to capture after closure of cells
Annual precipitation(mm)	≈53.02	≈24.76	≈24.76	≈24.76
Climate type (Köppen & Geiger)	Non-Arid/ wet climate	Semi-Arid /Moderate climate	Semi-Arid/moderate climate	Semi- Arid/ moderate climate
Mean annual temperature (°C)	22	32	32	32

### Emissions profiles of SWDs

#### Rawalpindi SWD emissions

Table 4 in Annexure illustrates the longitudinal trends in waste deposition and subsequent emissions from the Losar dumpsite between 2003 and 2052. During this period, the site received progressively increasing quantities of municipal solid waste (MSW), culminating in a total waste-in-place of 5.89 million megagrams (Mg) by 2025. Total gas emissions rose steadily to a peak of 43.81 Gg/year in 2025, accompanied by maximum methane and carbon dioxide outputs of 11.7 Gg/year and 32.1 Gg/year, respectively.

**Fig. 3:**
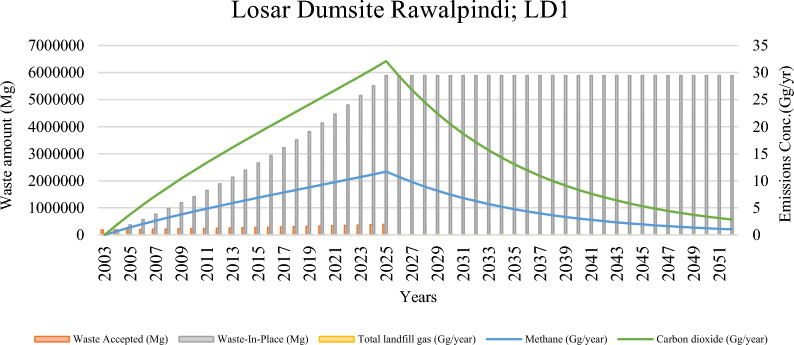
Landfill waste accumulation and emission trends at losar dumpsite (L1)

The period from 2016–2030 is identified as the most favourable timeframe for the effective capture of methane and landfill gas at this site (Fig. 3). The environmental footprints of this site are 7.6 Mt-CO_2_ eq for its whole lifespan (Table 3). Landfill gas (LFG) production exhibited a corresponding trend. Post-closure, a predictable decline in gas generation occurred, following a first-order decay function, which is consistent with IPCC modelling frameworks and empirical studies [53, 63]. Notably, waste input might cease in 2026 as per the plan, after which the landfill will transition into a closed state. But still, no plan for methane capturing or WtE program is reported.

**Table 3:** Summary of 50 years’ emissions profile of Rawalpindi and Lahore SWDs

Sites	Total Emissions (Gg))	Methane (Gg) → CO₂-eq	CO₂ (Gg)	NMOC (Gg)	Total Gg CO₂-eq	Mt-CO_2_ eq
LD1	958.5	255.97 → 7167.1	702.3	1.7	7869.4	7.9
MB1	5181.7	1383.9 → 38,747.2	3797	8.9	42,544.8	42.5
LD2	6152	1643 → 46,004	4508	11	50,510.0	50.5
LF3	2011.1	537 → 15,036	1474	3.46	16,510	16.5

Even after the cessation of waste acceptance, Losar is projected to emit residual methane for several decades. By 2052, methane generation is expected to decline to 1.03 Gg/year, approximately 9% of the peak value. Such prolonged emission tails have been observed at other landfills worldwide and reflect the slow degradation of recalcitrant organics under anaerobic conditions [11, 58].

Methane generation is directly proportional to the rate at which biodegradable substrates are metabolized by microbial communities under anaerobic conditions. The diverse waste fractions accepted at Losar, particularly food residues, contribute essential nutrients (e.g., carbohydrates, proteins, and lipids), enhancing microbial activity [24, 52, 54]. Rawalpindi’s typical diet, rich in wheat, barley, rice, pulses, and fats, supplies ample organic carbon, fostering methanogenesis. However, the heterogeneous composition of unsegregated waste, which includes plastics, rubber, metals, textiles, and inert materials, hinders complete biodegradation. These non-biodegradable fractions can dilute the effective biodegradability of the overall waste stream, potentially reducing methane yield [25, 47]. This duality in waste composition likely influenced the shape and intensity of the methane emission curve observed at Losar.

This sustained emission profile underscores the necessity for long-term post-closure monitoring and gas capture infrastructure. International guidelines recommend continued gas extraction until emissions fall below defined regulatory thresholds (e.g., US EPA NMOC limits), often requiring systems to remain operational for 20–30 years post-closure (US [67]). Furthermore, incorporating engineered biocovers or biofilters could enhance methane oxidation and reduce atmospheric release (Huber-Humer et al., 2008).

#### Lahore SWDs emissions

**Fig. 4:**
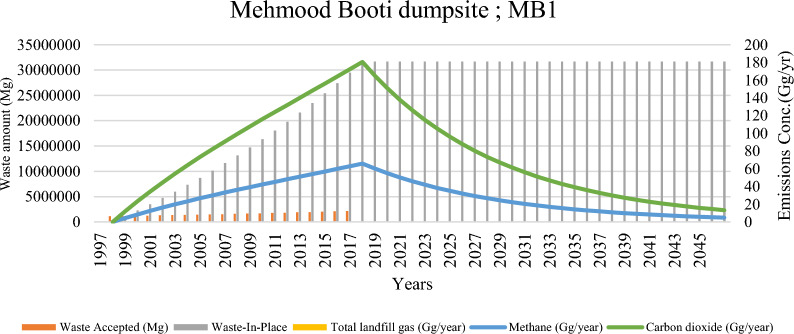
Landfill waste accumulation and emission trends at mehmood booti dumpsite (MB1)

**Fig. 5:**
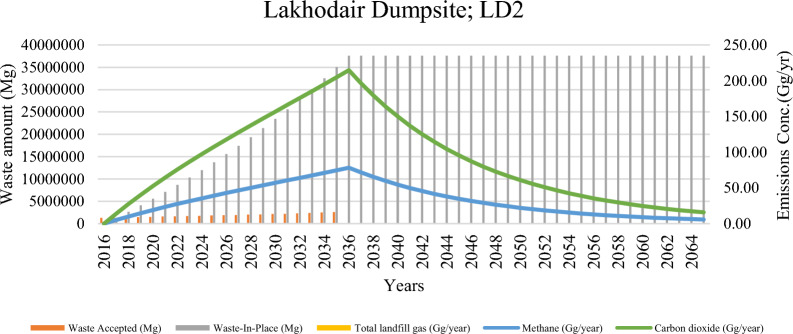
Landfill waste accumulation and emission trends at lakhodair dumpsite (LD2)

**Fig. 6:**
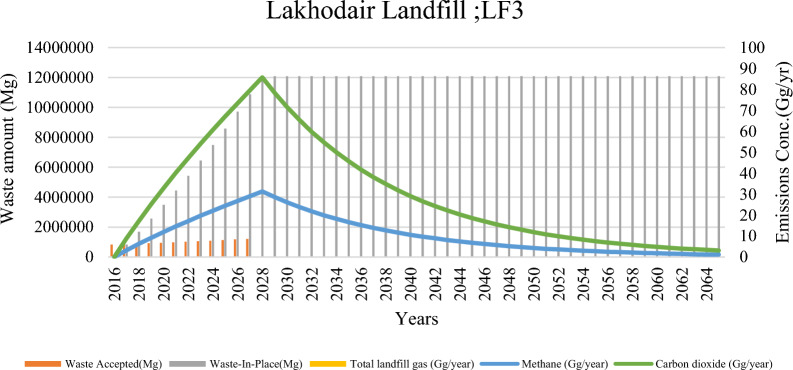
Landfill waste accumulation and emission trends at lakhodair landfill (LF3)

The emissions from the solid waste dumping sites (SWDs) in Lahore city are depicted in Figs. 4, 5, and 6. These figures show significant variability in total lifetime emissions, emission trajectories, and peak emission periods across different sites. Each of these factors has critical implications for strategies aimed at mitigating greenhouse gas (GHG) emissions. Additionally, Tables 5, 6, and 7 in the Annexure illustrate the model-computed longitudinal trends in waste deposition and the subsequent emissions from the key SWDs in Lahore, covering a period from the initial waste received through the following 50 years.

Mehmood Booti (MB1) (Fig. 4), despite being a closed site, exhibits a substantial legacy emission burden. It reached its peak methane emission of 65.83 Gg in 2017, shortly after closure. The peak aligns with the typical lag in maximum methane generation following the accumulation of biodegradable organic matter. Though emissions have gradually declined since then, MB1 continues to release more than 1 Gg/year of methane up to the mid-2040s. The environmental footprints of this site for the whole lifespan are 42.5 Mt-CO_2_ eq. (Table 3). 2006–2025 were recorded as significant years for methane capture. This prolonged emission tail presents a legacy climate risk, highlighting the importance of post-closure gas management, such as biocover systems or passive venting infrastructure.

Lakhodair Dumpsite (LD2) (Fig. 5), accounts for the highest cumulative methane emissions, releasing approximately 1,545 Gg of CH₄ over its operational and post-closure lifespan. The environmental footprints of this site for the whole lifespan are 50.5 Mt-CO_2_ eq. (Table 3). This surpasses both MB1 and LF3 due to its extended period of fresh municipal solid waste (MSW) intake (~ 4,000 tons/day) and the absence of engineered landfill gas (LFG) capture systems. Emission modeling shows a continual increase in methane output, culminating in a peak value of 78.25 Gg in 2036—the highest annual CH₄ emission recorded across all sites. 2024–2045 were recorded as significant years for methane capture. This projection establishes LD2 as the most critical site requiring immediate intervention through methane capture technologies before the 2030s.

Lakhodair Landfill (LF3), the first sanitary landfill in Pakistan, recorded a peak methane (CH₄) emission of 31.30 Gg during significant years for methane capture. Over its entire lifespan, the environmental footprint of this site amounts to 16.5 Mt of CO₂ equivalent. The Lahore Waste Management Company (LWMC) has implemented gas vents and capturing infrastructure to capture methane gas effectively. Currently, only two cells are operational, which limits the waste reception capacity and subsequently restricts the potential for methane generation.

Cumulative emissions from Lahore’s solid waste disposal (SWD) systems were calculated at approximately 133,446 Gg, equivalent to 108 Mt CO₂-eq, with contributions comprising 26% methane, 73% carbon dioxide (CO₂), and 0.2% non-methane organic compounds (NMOCs). In contrast, Rawalpindi’s SWD systems generated 958 Gg (or 7.9 Mt CO₂-eq) over their operational life, exhibiting a similar emissions profile. Two unmanaged Lahore sites—LD2 (1643 Gg CH₄) and MB1 (1383.9 Gg CH₄)—emerged as the most significant methane emitters across both cities. The temporal patterns highlight the unique emission risk profiles. For the LD2 profile, there is a rising methane curve with a peak expected in 2036, indicating an urgent need for methane capture infrastructure. The MB1 profile shows a post-peak decline followed by a long emission tail, suggesting the necessity for legacy emission monitoring and mitigation strategies. Meanwhile, the LF3 profile exhibits an early peak that gradually declines, accompanied by higher emissions of non-methane organic compounds (NMOC), which raises concerns about urban health implications [35]. Cumulatively, Lahore’s total methane emissions surpass those of Rawalpindi, reflecting the city’s larger population, higher waste generation rates, and lifestyle-related waste patterns. Past studies, such as by Munir et al. [39], have reported CO₂ and CH₄ emissions totaling over 50 million kg in Ravi Town alone, aligning with the current study’s estimates and confirming the magnitude of the problem.

The data call for an integrated waste management approach encompassing methane mitigation, NMOC reduction, and long-term emission monitoring for urban environmental sustainability.

The absence of environmental buffers, proper zoning, and engineered barriers in cities like Lahore results in land-use conflicts, odor nuisance, vector-borne diseases, and the devaluation of surrounding property [30]. Moreover, large-scale emissions contribute to atmospheric photochemical reactions, increasing the burden of ground-level ozone (O₃) and smog, further endangering respiratory health, particularly in children and the elderly (WHO [68]).

The environmental implications of these findings are substantial. Lahore, already ranked highest in global air pollution indices, faces compounded challenges from landfill-based emissions. NMOCs have direct consequences for public health, contributing to ozone formation and respiratory disorders [9]. Addressing these emissions necessitates the adoption of advanced landfill management strategies. Based on literature; Methane capture and utilization technologies, optimized landfill covers, and engineered biogas recovery systems [51], waste minimization through enhanced segregation, recycling, and composting could substantially reduce organic load and subsequent methane generation (Thomson et al., 2022) could be adopted for both cities. Proven technologies such as Vertical and horizontal gas extraction wells, Gas flaring units, and Biogas-to-electricity plants have been successfully implemented in countries with similar waste streams and climatic conditions (e.g., India, Brazil). For example, India’s Ghazipur and Bhalswa sites are now equipped with gas capture systems that mitigate over 50 Gg CH₄/year, which parallels the emissions estimated in this study (Kaushal & Sharma 2016).

Comparing emissions across regions remains complex due to heterogeneity in waste composition, climate, and landfill management. The limited availability of emission studies from open dumpsites in developing countries underscores the need for more empirical research to support evidence-based waste policy formulation (IPCC [26]b; Kaza et al., 2018). The scale and intensity of emissions, particularly at Lakhodair Dumpsite (LD2), suggest strong feasibility for landfill gas recovery and utilization systems. Lahore’s waste profile—high in organic fraction also makes it a strong candidate for anaerobic digestion (AD) and waste-to-energy (WTE) projects.MSW in Rawalpindi and Lahore could generate 49 MW and 341 MW of electricity daily, respectively, if supported by suitable policies and technologies [24]. AD technology could be deployed at transfer stations or decentralized waste processing centers to pre-treat organics, reducing landfill loading and methane generation.

Both cities’ data represent a major untapped opportunity for climate mitigation, renewable energy production, and improved urban environmental health. By translating emissions data into targeted interventions—both technological and regulatory—the city can move toward a low-carbon waste management future while addressing critical public health vulnerabilities.

#### Results: leachate generation and sensitivity analysis

##### Leachate generation

**Fig. 7:**
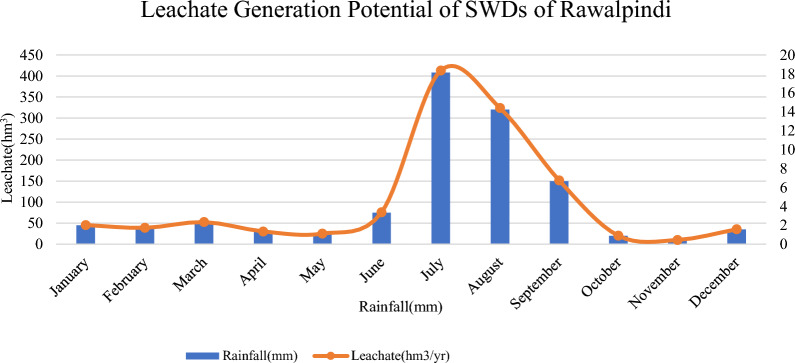
Leachate generation potential of SWDs of Rawalpindi

**Fig. 8:**
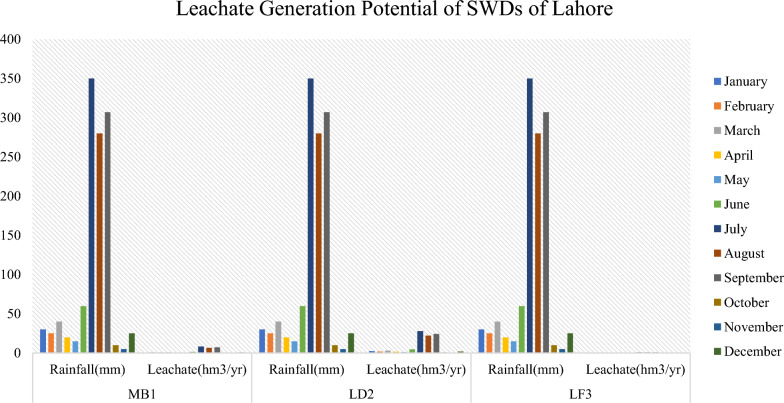
Leachate generation potential of SWDs of Lahore

Leachate generation exhibited a strong positive correlation with rainfall patterns across the study period (Figs. 7 & 8). Rainfall displayed marked monthly variability, peaking at 408 mm in July, consistent with regional monsoon dynamics reported by Mahmood et al. (2019) and Khan et al. (2022). The monsoon months (July–September) received 320–408 mm of rainfall, whereas the dry season (November–February) recorded only 10–45 mm. This seasonal variability directly influenced leachate volumes, with maximum generation reaching 18.36 million m^3^ in July and minimum levels of 0.45 million m^3^ in November, confirming rainfall as the dominant driver of leachate formation, as also observed by Aziz et al. (2020). The relationship between rainfall and leachate generation reflects a multiplier effect (Chen et al., 2022), wherein infiltration through waste layers amplifies the rainfall input due to waste porosity, inadequate cover, and hydraulic connectivity. The resulting leachate is a complex effluent containing ammonia, heavy metals, volatile organics, and pathogens, posing substantial risks to groundwater and soil quality ([19], [28]). Alao [8] reported comparable contamination trends in Nigerian dumpsites, where safe groundwater was only available below 23 m, indicating extensive leachate percolation. Such findings underscore the urgent need for seasonal leachate management strategies, including optimized cover application, treatment capacity adjustments, and real-time monitoring during high rainfall periods ([57], [55]).

The sustained high leachate concentrations observed in the present study highlight potential contamination risks for underlying aquifers and adjacent soils, emphasizing the necessity of integrated landfill design and operational control to mitigate long-term environmental degradation.

##### Sensitivity analysis

**Fig. 9:**
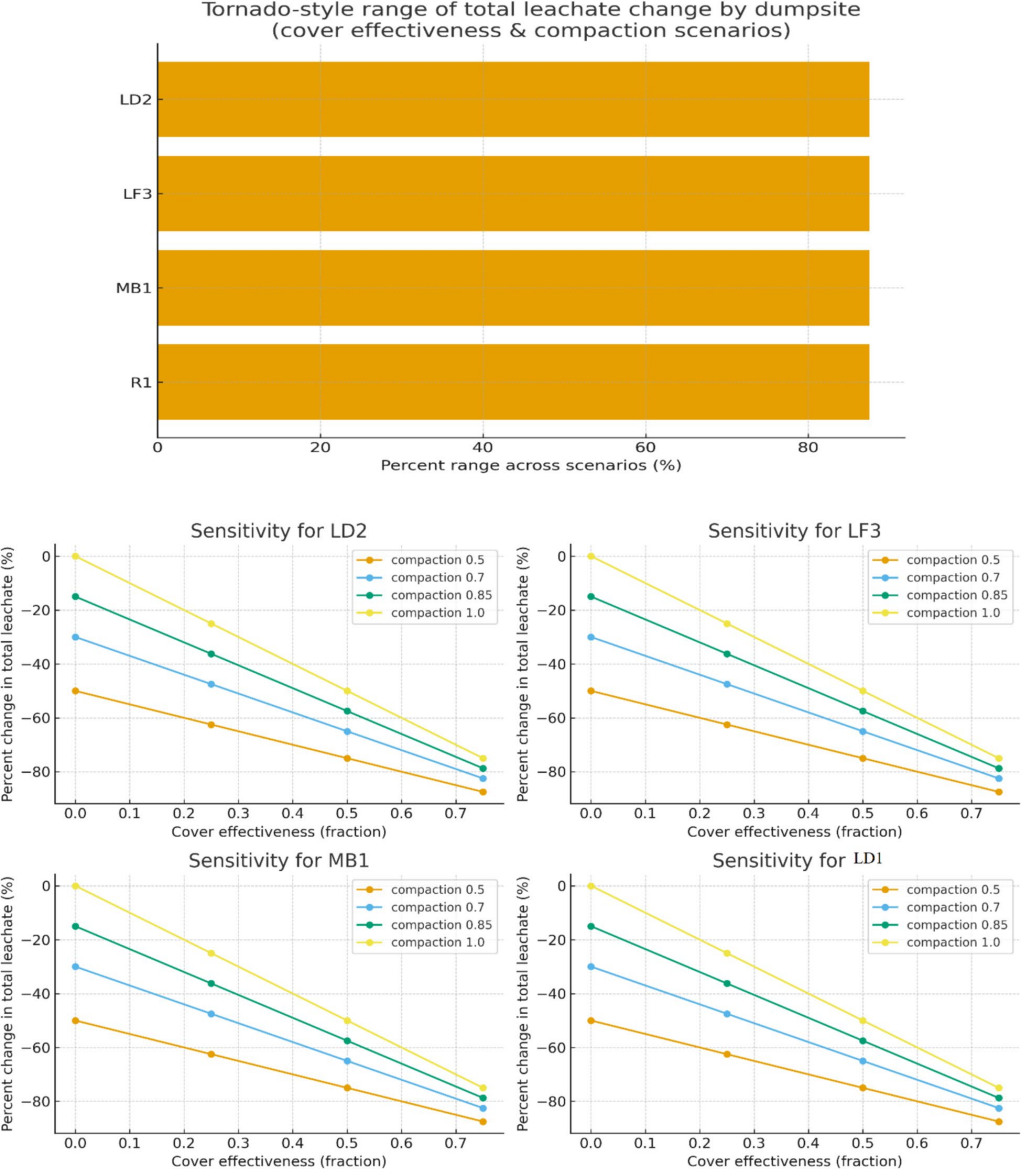
Sensitivity analysis of leachate generation from all studied sites

The sensitivity analysis (Fig. 9) demonstrated that both surface cover efficiency and waste compaction substantially influence leachate generation. Increasing cover application to 50% combined with a compaction factor of 0.70 reduced total leachate output by 35–45% relative to baseline conditions. The tornado-style plot illustrates that dumpsites R1 and LD2 exhibited the highest sensitivity, with changes exceeding 40%, indicating that site-specific operational improvements could yield significant reductions in leachate flux.

These results align with previous landfill hydrology studies that highlight the dominant role of operational parameters in controlling moisture infiltration [17, 28, 42]. Enhanced daily or seasonal cover and higher compaction densities effectively limit infiltration and percolation, thereby lowering both the quantity and pollutant load of generated leachate [18], [1]. Al-Yaqout and Hamoda [6] and Lou and Nair [34] similarly observed that intermediate cover systems can reduce infiltration by up to 60–70% per cent, particularly in tropical environments.

While the simplified multiplicative model used here does not capture all hydrological complexities—such as evapotranspiration dynamics or preferential flow pathways—it provides a first-order quantitative estimate of leachate response to operational modifications. Comparable parametric approaches have been effectively employed by Beaven [15] and Abushammala et al. [2] to assess settlement and leachate variability under different landfill management regimes.

Future studies should integrate process-based hydrological models such as SWAT, HYDRUS, or HELP to simulate unsaturated flow and moisture redistribution in greater detail [33, 59]. Nonetheless, the current analysis demonstrates that operational optimization—through increased compaction and improved cover efficiency—can substantially mitigate leachate generation, supporting the broader consensus that effective management practices are as critical as engineered controls in achieving sustainable landfill performance.

The seasonal magnitudes and pollutant loads reported here are consistent with findings from comparable South Asian sites. Field studies from Lahore indicate pronounced monsoon peaks in leachate volume and high organic and inorganic pollutant concentrations [10], and detailed leachate characterizations from Dhaka landfills (Matuail, Amin Bazar) report similar seasonal spikes in BOD, COD, NH₄⁺ and heavy metals during monsoon months [5, 41]. Studies from India and Sri Lanka likewise document strong monsoon-driven increases in leachate flux and groundwater impacts [38, 56], while national methane inventories and city-scale studies for Pakistan and India provide congruent orders of magnitude for CH₄ emissions from unmanaged disposal sites [49], LandGEM applications). Collectively, these South Asian studies validate the direction and approximate magnitude of our emission and leachate outputs and support the recommendation for seasonal scaling of leachate treatment and intensified monitoring during peak rainfall.

## Conclusions & recommendations

Landfill sites in Lahore and Rawalpindi are major sources of gas emissions, releasing a total of 13,344 Gg (109 Mt-CO_2_ Equ.) and 958.5 Gg (7.8 Mt-CO_2_ Equ.) respectively, over their 50-year operational lifespan. The lack of scientifically sound treatment methods contributes to increased greenhouse gas (GHG) emissions from dumpsites and landfills, which generate significant leachate that poses a threat to groundwater and soil. The growing population, urbanization, and changing lifestyles further exacerbate future waste quality and emissions. This research establishes a quantitative baseline for capturing landfill gas (LFG) to support waste-to-energy (WtE) initiatives, providing a strategy to reduce emissions and combat climate change.

Future studies should focus on the long-term effects of climate change on leachate generation, real-time monitoring systems, and predictive models to better understand and manage these complex interactions. Key enabling actions include:

- Public–private investment schemes for WtE facilities (as piloted in Karachi)
- Regulatory approvals and feed-in tariffs for electricity from biogas under NEPRA’s renewable energy policy
- Pilot-scale deployment of CALMIM or IPCC Tier II hybrid systems for better methane prediction and recovery design

### LandGem model limitations and way forward

The U.S. EPA’s Landfill Gas Emission Model (LandGEM v3.1) was applied to estimate methane and NMOC emissions. However, its assumptions—such as a single first-order decay process, fully anaerobic conditions, and default parameter values (k and L₀)—may not accurately represent the characteristics of unmanaged open dumps in Pakistan. These sites exhibit heterogeneous waste composition, partial aeration, moisture pulses from monsoon events, and an absence of gas collection systems. Consequently, LandGEM may overestimate emissions under default anaerobic conditions or underestimate them when localized wet zones and surface hotspots dominate gas release. To address this, locally derived waste composition data and sensitivity analyses for k and L₀ were incorporated, and field verification using flux chamber and gas well sampling is recommended.

## Data Availability

No datasets were generated or analysed during the current study.

## Annexure 1 (emissions results).

See annexure Tables 4, 5, 6, 7

**Table 4:** Waste and emissions estimates of Losar Dumpsite (L1), Rawalpindi

Sr. No	Year	Waste accepted (Mg)	Waste-in-place (Mg)	Total landfill gas (Gg/year)	Methane (Gg/year)	Carbon dioxide (Gg/year)	NMOC (Gg/year)
1	2003	182,119	0	0	0	0	0
2	2004	188,493	182,119	2.662261	0.711	1.950817	0.004584
3	2005	195,091	370,612	5.185979	1.385	3.800115	0.00893
4	2006	201,919	565,703	7.589877	2.027	5.561613	0.013069
5	2007	208,986	767,622	9.885187	2.64	7.243541	0.017021
6	2008	216,300	976,608	12.08689	3.228	8.856875	0.020812
7	2009	223,871	1,192,908	14.20996	3.795	10.41259	0.024468
8	2010	231,706	1,416,779	16.25439	4.341	11.91069	0.027988
9	2011	239,816	1,648,485	18.24266	4.872	13.36763	0.031412
10	2012	248,210	1,888,301	20.17477	5.388	14.78341	0.034738
11	2013	256,897	2,136,511	22.06568	5.893	16.16901	0.037994
12	2014	265,889	2,393,408	23.91916	6.388	17.52717	0.041186
13	2015	275,195	2,659,297	25.74642	6.876	18.86613	0.044332
14	2016	284,826	2,934,492	27.55121	7.358	20.18863	0.04744
15	2017	294,795	3,219,318	29.34103	7.836	21.50015	0.050522
16	2018	305,113	3,514,113	31.12336	8.312	22.80618	0.053591
17	2019	315,792	3,819,226	32.90195	8.787	24.10947	0.056653
18	2020	326,845	4,135,018	34.68428	9.263	25.4155	0.059722
19	2021	338,284	4,461,863	36.47409	9.741	26.72702	0.062804
20	2022	350,124	4,800,147	38.27514	10.222	28.04677	0.065905
21	2023	362,379	5,150,271	40.09492	10.708	29.38024	0.069039
22	2024	375,062	5,512,650	41.9409	11.201	30.73292	0.072217
23	2025	388,189	5,887,712	43.80935	11.7	32.10206	0.075434
24	2026	0	5,887,712	40.03875	10.693	29.33909	0.068942
25	2027	0	5,887,712	36.59391	9.773	26.81482	0.06301
26	2028	0	5,887,712	33.44488	8.932	24.50731	0.057588
27	2029	0	5,887,712	30.56545	8.163	22.39736	0.05263
28	2030	0	5,887,712	27.93314	7.46	20.46849	0.048097
29	2031	0	5,887,712	25.52924	6.818	18.70699	0.043958
30	2032	0	5,887,712	23.33129	6.231	17.0964	0.040174
31	2033	0	5,887,712	21.3243	5.695	15.62575	0.036718
32	2034	0	5,887,712	19.48954	5.205	14.2813	0.033559
33	2035	0	5,887,712	17.81206	4.757	13.05209	0.03067
34	2036	0	5,887,712	16.27686	4.347	11.92715	0.028027
35	2037	0	5,887,712	14.87646	3.973	10.90098	0.025615
36	2038	0	5,887,712	13.59588	3.631	9.962613	0.02341
37	2039	0	5,887,712	12.42763	3.319	9.106558	0.021399
38	2040	0	5,887,712	11.35673	3.033	8.321841	0.019555
39	2041	0	5,887,712	10.37945	2.772	7.605718	0.017872
40	2042	0	5,887,712	9.484537	2.533	6.949958	0.016331
41	2043	0	5,887,712	8.668261	2.315	6.351817	0.014926
42	2044	0	5,887,712	7.923127	2.116	5.805808	0.013643
43	2045	0	5,887,712	7.241648	1.934	5.306443	0.012469
44	2046	0	5,887,712	6.62008	1.768	4.850978	0.011399
45	2047	0	5,887,712	6.047188	1.615	4.431181	0.010413
46	2048	0	5,887,712	5.526718	1.476	4.049798	0.009516
47	2049	0	5,887,712	5.051181	1.349	3.70134	0.008698
48	2050	0	5,887,712	4.616832	1.233	3.383063	0.00795
49	2051	0	5,887,712	4.219926	1.127	3.092224	0.007266
50	2052	0	5,887,712	3.856721	1.03	2.826079	0.006641

**Table 5:** Waste and emissions estimates of Mehmood Boti Dumpsite (MB1)

Sr.no	Year	Waste accepted (Mg)	Waste-in-place (Mg)	Total landfill gas (Gg/year)	Methane (Gg/year)	Carbon dioxide (Gg/year)	NMOC (Gg/year)
1	1997	1,119,536	0.00	0	0	0	0
2	1998	1,158,722	1,119,536.00	16.355	4.368	11.985	0.028
3	1999	1,199,278	2,278,258.00	31.876	8.513	23.358	0.055
4	2000	1,241,245	3,477,536.00	46.655	12.460	34.187	0.080
5	2001	1,284,692	4,718,781.00	60.775	16.231	44.534	0.105
6	2002	1,329,656	6,003,473.00	74.311	19.846	54.453	0.128
7	2003	1,376,192	7,333,129.00	87.342	23.326	64.001	0.150
8	2004	1,424,361	8,709,321.00	99.930	26.688	73.226	0.172
9	2005	1,474,215	10,133,682.00	112.141	29.949	82.173	0.193
10	2006	1,525,811	11,607,897.00	124.025	33.123	90.882	0.214
11	2007	1,579,215	13,133,708.00	135.644	36.226	99.396	0.234
12	2008	1,634,480	14,712,923.00	147.038	39.269	107.745	0.253
13	2009	1,691,685	16,347,403.00	158.264	42.267	115.971	0.273
14	2010	1,750,897	18,039,088.00	169.359	45.230	124.101	0.292
15	2011	1,812,179	19,789,985.00	180.360	48.168	132.162	0.311
16	2012	1,875,603	21,602,164.00	191.312	51.093	140.187	0.329
17	2013	1,941,248	23,477,767.00	202.249	54.014	148.202	0.348
18	2014	2,009,192	25,419,015.00	213.202	56.939	156.227	0.367
19	2015	2,079,513	27,428,207.00	224.207	59.878	164.291	0.386
20	2016	2,152,318	29,507,720.00	235.290	62.838	172.413	0.405
21	2017	0	31,660,038.00	246.486	65.828	180.617	0.424
22	2018	0	31,660,038.00	225.270	60.162	165.070	0.388
23	2019	0	31,660,038.00	205.881	54.984	150.863	0.355
24	2020	0	31,660,038.00	188.163	50.252	137.880	0.324
25	2021	0	31,660,038.00	171.969	45.927	126.013	0.296
26	2022	0	31,660,038.00	157.167	41.974	115.167	0.271
27	2023	0	31,660,038.00	143.639	38.361	105.254	0.247
28	2024	0	31,660,038.00	131.275	35.059	96.194	0.226
29	2025	0	31,660,038.00	119.978	32.042	87.916	0.207
30	2026	0	31,660,038.00	109.651	29.284	80.348	0.189
31	2027	0	31,660,038.00	100.215	26.764	73.434	0.173
32	2028	0	31,660,038.00	91.588	24.460	67.113	0.158
33	2029	0	31,660,038.00	83.706	22.355	61.337	0.144
34	2030	0	31,660,038.00	76.502	20.431	56.058	0.132
35	2031	0	31,660,038.00	69.915	18.672	51.232	0.120
36	2032	0	31,660,038.00	63.898	17.065	46.822	0.110
37	2033	0	31,660,038.00	58.397	15.596	42.792	0.101
38	2034	0	31,660,038.00	53.373	14.254	39.110	0.092
39	2035	0	31,660,038.00	48.778	13.027	35.743	0.084
40	2036	0	31,660,038.00	44.581	11.906	32.667	0.077
41	2037	0	31,660,038.00	40.743	10.881	29.855	0.070
42	2038	0	31,660,038.00	37.238	9.945	27.287	0.064
43	2039	0	31,660,038.00	34.033	9.089	24.938	0.059
44	2040	0	31,660,038.00	31.105	8.307	22.792	0.054
45	2041	0	31,660,038.00	28.427	7.592	20.831	0.049
46	2042	0	31,660,038.00	25.979	6.938	19.036	0.045
47	2043	0	31,660,038.00	23.743	6.341	17.398	0.041
48	2044	0	31,660,038.00	21.699	5.795	15.900	0.037
49	2045	0	31,660,038.00	19.830	5.296	14.531	0.034
50	2046	0	31,660,038.00	18.127	4.841	13.283	0.031

**Table 6:** Waste and emissions estimates of Lakhodair Dumpsite (LD2)

Sr. no	Year	Waste accepted (Mg)	Waste-in-place (Mg)	Total landfill gas (Gg/year)	Methane (Gg/year)	Carbon dioxide (Gg/year)	NMOC (Gg/year)
1	2016	1,324,503	0	0	0	0	0
2	2017	1,370,861	1,324,503	19.351	5.168	14.17978	0.03332
3	2018	1,419,295	2,695,364	37.71349	10.072	27.63521	0.064938
4	2019	1,469,424	4,114,659	55.20353	14.743	40.45134	0.095054
5	2020	1,521,307	5,584,083	71.91848	19.207	52.69951	0.123835
6	2021	1,575,002	7,105,390	87.9557	23.49	64.45105	0.151449
7	2022	1,630,583	8,680,392	103.3976	27.614	75.76634	0.178038
8	2023	1,688,124	10,310,975	118.3189	31.599	86.70025	0.203731
9	2024	1,747,702	11,999,099	132.7985	35.466	97.31039	0.228663
10	2025	1,809,405	13,746,801	146.9036	39.233	107.6462	0.25295
11	2026	1,873,329	15,556,206	160.6942	42.916	117.7514	0.276696
12	2027	1,939,577	17,429,535	174.2339	46.532	127.6729	0.30001
13	2028	2,008,259	19,369,112	187.5714	50.094	137.4462	0.322975
14	2029	2,079,491	21,377,371	200.7704	53.619	147.118	0.345702
15	2030	2,153,397	23,456,862	213.8683	57.117	156.7157	0.368255
16	2031	2,230,107	25,610,259	226.925	60.604	166.2832	0.390737
17	2032	2,309,757	27,840,366	239.9741	64.089	175.8452	0.413206
18	2033	2,392,489	30,150,123	253.0645	67.585	185.4374	0.435746
19	2034	2,478,453	32,542,612	266.2373	71.103	195.09	0.458428
20	2035	2,567,897	35,021,065	279.5336	74.654	204.8331	0.481323
21	2036	0	37,588,962	292.991	78.248	214.6942	0.504495
22	2037	0	37,588,962	267.7725	71.513	196.2149	0.461072
23	2038	0	37,588,962	244.7258	65.358	179.327	0.421388
24	2039	0	37,588,962	223.6636	59.733	163.8933	0.385121
25	2040	0	37,588,962	204.4137	54.592	149.7877	0.351975
26	2041	0	37,588,962	186.8188	49.893	136.8947	0.321679
27	2042	0	37,588,962	170.7404	45.599	125.113	0.293994
28	2043	0	37,588,962	156.0437	41.674	114.3437	0.268688
29	2044	0	37,588,962	142.6125	38.087	104.5018	0.245561
30	2045	0	37,588,962	130.3384	34.809	95.50774	0.224427
31	2046	0	37,588,962	119.1202	31.813	87.28741	0.205111
32	2047	0	37,588,962	108.8681	29.075	79.77498	0.187458
33	2048	0	37,588,962	99.49965	26.573	72.91008	0.171326
34	2049	0	37,588,962	90.93249	24.285	66.63235	0.156575
35	2050	0	37,588,962	83.10671	22.195	60.89788	0.1431
36	2051	0	37,588,962	75.95493	20.285	55.65728	0.130785
37	2052	0	37,588,962	69.41723	18.539	50.86667	0.119528
38	2053	0	37,588,962	63.44118	16.943	46.48762	0.109238
39	2054	0	37,588,962	57.98186	15.485	42.48721	0.099838
40	2055	0	37,588,962	52.99059	14.152	38.82977	0.091243
41	2056	0	37,588,962	48.42993	12.934	35.48786	0.08339
42	2057	0	37,588,962	44.26242	11.821	32.43405	0.076214
43	2058	0	37,588,962	40.45438	10.804	29.64364	0.069658
44	2059	0	37,588,962	36.9721	9.874	27.09194	0.063661
45	2060	0	37,588,962	33.78937	9.024	24.75974	0.058181
46	2061	0	37,588,962	30.87998	8.247	22.62783	0.053172
47	2062	0	37,588,962	28.22146	7.537	20.67976	0.048594
48	2063	0	37,588,962	25.7951	6.889	18.9018	0.044416
49	2064	0	37,588,962	23.57467	6.296	17.27475	0.040593
50	2065	0	37,588,962	21.54521	5.754	15.78763	0.037098

**Table 7:** Waste and emissions estimates of Lakhodair landfill (LF3)

Sr.no	Year	Waste accepted (Mg)	Waste-in-place (Mg)	Total landfill gas (Gg/year)	Methane (Gg/year)	Carbon dioxide (Gg/year)	NMOC (Gg/year)
1	2016	**827,815**	0	0	0	0	0
2	2017	856,788	827,815	12.1	3.2	8.9	0.02
3	2018	886,776	1,684,603	23.6	6.3	17.3	0.04
4	2019	917,813	2,571,379	34.5	9.2	25.3	0.06
5	2020	949,936	3,489,192	44.9	12.0	32.9	0.08
6	2021	983,184	4,439,128	54.9	14.7	40.3	0.09
7	2022	1,017,595	5,422,312	64.6	17.2	47.3	0.11
8	2023	1,053,211	6,439,907	73.9	19.7	54.1	0.13
9	2024	1,090,074	7,493,118	82.9	22.1	60.8	0.14
10	2025	1,128,226	8,583,192	91.7	24.5	67.2	0.16
11	2026	1,167,714	9,711,418	100.3	26.8	73.5	0.17
12	2027	1,208,584	10,879,132	108.7	29.0	79.7	0.19
13	2028	0	12,087,716	117.0	31.3	85.8	0.20
14	2029	0	12,087,716	107.0	28.6	78.4	0.18
15	2030	0	12,087,716	97.7	26.1	71.6	0.17
16	2031	0	12,087,716	89.3	23.9	65.5	0.15
17	2032	0	12,087,716	81.6	21.8	59.8	0.14
18	2033	0	12,087,716	74.6	19.9	54.7	0.13
19	2034	0	12,087,716	68.2	18.2	50.0	0.12
20	2035	0	12,087,716	62.3	16.6	45.7	0.11
21	2036	0	12,087,716	57.0	15.2	41.7	0.10
22	2037	0	12,087,716	52.1	13.9	38.1	0.09
23	2038	0	12,087,716	47.6	12.7	34.9	0.08
24	2039	0	12,087,716	43.5	11.6	31.9	0.07
25	2040	0	12,087,716	39.7	10.6	29.1	0.07
26	2041	0	12,087,716	36.3	9.7	26.6	0.06
27	2042	0	12,087,716	33.2	8.9	24.3	0.06
28	2043	0	12,087,716	30.3	8.1	22.2	0.05
29	2044	0	12,087,716	27.7	7.4	20.3	0.05
30	2045	0	12,087,716	25.3	6.8	18.6	0.04
31	2046	0	12,087,716	23.2	6.2	17.0	0.04
32	2047	0	12,087,716	21.2	5.7	15.5	0.04
33	2048	0	12,087,716	19.3	5.2	14.2	0.03
34	2049	0	12,087,716	17.7	4.7	13.0	0.03
35	2050	0	12,087,716	16.2	4.3	11.8	0.03
36	2051	0	12,087,716	14.8	3.9	10.8	0.03
37	2052	0	12,087,716	13.5	3.6	9.9	0.02
38	2053	0	12,087,716	12.3	3.3	9.0	0.02
39	2054	0	12,087,716	11.3	3.0	8.3	0.02
40	2055	0	12,087,716	10.3	2.8	7.5	0.02
41	2056	0	12,087,716	9.4	2.5	6.9	0.02
42	2057	0	12,087,716	8.6	2.3	6.3	0.01
43	2058	0	12,087,716	7.9	2.1	5.8	0.01
44	2059	0	12,087,716	7.2	1.9	5.3	0.01
45	2060	0	12,087,716	6.6	1.8	4.8	0.01
46	2061	0	12,087,716	6.0	1.6	4.4	0.01
47	2062	0	12,087,716	5.5	1.5	4.0	0.01
48	2063	0	12,087,716	5.0	1.3	3.7	0.01
49	2064	0	12,087,716	4.6	1.2	3.4	0.01
50	2065	0	12,087,716	4.2	1.1	3.1	0.01

## Annexure 2 sensitivity analysis

**Table:**

Dumpsite	Baseline Leachate (hm^3^/yr)	Scenario Leachate (hm^3^/yr)	Cover Effectiveness (fraction)	Compaction Factor	Percent Change (%)
LD2	183.168	91.584	0	0.5	-50
LD2	183.168	128.218	0	0.7	-30
LD2	183.168	155.693	0	0.85	-15
LD2	183.168	183.168	0	1	0
LD2	183.168	68.688	0.25	0.5	-62.5
LD2	183.168	96.163	0.25	0.7	-47.5
LD2	183.168	116.77	0.25	0.85	-36.25
LD2	183.168	137.376	0.25	1	-25
LD2	183.168	45.792	0.5	0.5	-75
LD2	183.168	64.109	0.5	0.7	-65
LD2	183.168	77.846	0.5	0.85	-57.5
LD2	183.168	91.584	0.5	1	-50
LD2	183.168	22.896	0.75	0.5	-87.5
LD2	183.168	32.054	0.75	0.7	-82.5
LD2	183.168	38.923	0.75	0.85	-78.75
LD2	183.168	45.792	0.75	1	-75
LF3	6.912	3.456	0	0.5	-50
LF3	6.912	4.838	0	0.7	-30
LF3	6.912	5.875	0	0.85	-15
LF3	6.912	6.912	0	1	0
LF3	6.912	2.592	0.25	0.5	-62.5
LF3	6.912	3.629	0.25	0.7	-47.5
LF3	6.912	4.406	0.25	0.85	-36.25
LF3	6.912	5.184	0.25	1	-25
LF3	6.912	1.728	0.5	0.5	-75
LF3	6.912	2.419	0.5	0.7	-65
LF3	6.912	2.938	0.5	0.85	-57.5
LF3	6.912	3.456	0.5	1	-50
LF3	6.912	0.864	0.75	0.5	-87.5
LF3	6.912	1.21	0.75	0.7	-82.5
LF3	6.912	1.469	0.75	0.85	-78.75
LF3	6.912	1.728	0.75	1	-75
MB1	55.296	27.648	0	0.5	-50
MB1	55.296	38.707	0	0.7	-30
MB1	55.296	47.002	0	0.85	-15
MB1	55.296	55.296	0	1	0
MB1	55.296	20.736	0.25	0.5	-62.5
MB1	55.296	29.03	0.25	0.7	-47.5
MB1	55.296	35.251	0.25	0.85	-36.25
MB1	55.296	41.472	0.25	1	-25
MB1	55.296	13.824	0.5	0.5	-75
MB1	55.296	19.354	0.5	0.7	-65
MB1	55.296	23.501	0.5	0.85	-57.5
MB1	55.296	27.648	0.5	1	-50
MB1	55.296	6.912	0.75	0.5	-87.5
MB1	55.296	9.677	0.75	0.7	-82.5
MB1	55.296	11.75	0.75	0.85	-78.75
MB1	55.296	13.824	0.75	1	-75
LD1	106.74	53.37	0	0.5	-50
LD1	106.74	74.718	0	0.7	-30
LD1	106.74	90.729	0	0.85	-15
LD1	106.74	106.74	0	1	0
LD1	106.74	40.028	0.25	0.5	-62.5
LD1	106.74	56.038	0.25	0.7	-47.5
LD1	106.74	68.047	0.25	0.85	-36.25
LD1	106.74	80.055	0.25	1	-25
LD1	106.74	26.685	0.5	0.5	-75
LD1	106.74	37.359	0.5	0.7	-65
LD1	106.74	45.364	0.5	0.85	-57.5
LD1	106.74	53.37	0.5	1	-50
LD1	106.74	13.342	0.75	0.5	-87.5
LD1	106.74	18.68	0.75	0.7	-82.5
LD1	106.74	22.682	0.75	0.85	-78.75
LD1	106.74	26.685	0.75	1	-75

## Abbreviations

MSW: Municipal solid waste
SWD: Solid waste dumpsite
Gg: Gigagram
hm^3^/yr: Hectometer cubed per year
GHGs: Greenhouse gases
IPCC: Intergovernmental panel on climate change
GCMs: Global circulation models
UNFCCC: United nations framework convention on climate change
RWMC: Rawalpindi waste management company
WASA: Water and sanitation agency
EPD: Environmental protection department
LWMC: Lahore waste management company

## References

[1] Abdallah, M., Shanableh, A., Shabib, A., & Adghim, M. (2018). Financial feasibility of waste to energy strategies in the United Arab Emirates. Waste Management, 82, 207-219. 5. 10.1016/j.wasman.2018.10.029 30509583

[2] Abushammala MF, Basri NEA, Younes MK. Leachate generation and characteristics from Malaysian landfills: influence of operational practices. Environ Monit Assess. 2016;188(5):275.27059034

[3] Ahmed, Z. F., Alnuaimi, A. K., Askri, A., & Tzortzakis, N. (2021). Evaluation of Lettuce (Lactuca sativa L.) production under hydroponic system: Nutrient solution derived from fish waste vs. Inorganic nutrient solution. Horticulturae, 7(9), 292.

[4] Akmal T, Jamil F. Assessing health damages from improper disposal of solid waste in metropolitan Islamabad–Rawalpindi, Pakistan. Sustainability. 2021;13(5):2717.

[5] Akter S, Shammi M, Jolly YN, Sakib AA, Rahman MM, Tareq SM. Characterization and photodegradation pathway of the leachate of Matuail sanitary landfill site, Dhaka South City Corporation, Bangladesh. Heliyon. 2021;7(9):e07924. 10.1016/j.heliyon.2021.e07924. 34527825 10.1016/j.heliyon.2021.e07924 PMC8429108

[6] Al-Yaqout AF, Hamoda MF. Evaluation of landfill leachate in arid climate—a case study. Environ Int. 2003;29(5):593–600. 12742402 10.1016/S0160-4120(03)00018-7

[7] Alam P, Ahmade K, Iqbal MT. Greenhouse gas emission potential from municipal solid waste in Dhaka. Energy Environ Res. 2013;3(2):125–32.

[8] Alao JO. Impacts of open dumpsite leachates on soil and groundwater quality. Groundw Sustain Dev. 2023;20:100877.

[9] Alyasi HS, Isaifan R. A review on pollution emissions and impact from waste treatment and disposal facilities. J Environ Toxicol Stud. 2018;2(1):1–9.

[10] Azam S, Farid S, Nawaz M. Seasonal variation in leachate composition and environmental impacts at Lahore municipal solid waste landfill, Pakistan. Environ Monit Assess. 2020;192(11):694. 10.1007/s10661-020-08655-3.33037931

[11] Barlaz MA, Chanton JP, Green RB. Controls on landfill gas collection efficiency: instantaneous and lifetime performance. J Air Waste Manag Assoc. 2010;59(12):1399–404. 10.3155/1047-3289.59.12.1399 20066905

[12] Batool SA, Chuadhry MN. The impact of municipal solid waste treatment methods on greenhouse gas emissions in Lahore, Pakistan. Waste Manag. 2009;29(1):63–9. 18387288 10.1016/j.wasman.2008.01.013

[13] Batool SA, Nawaz M. Municipal solid waste management in Lahore city district, Pakistan. Waste Manag. 2009;29(6):1971–81. 19157840 10.1016/j.wasman.2008.12.016

[14] Batool, S. A., Chaudhry, N., & Majeed, K. (2008). Economic potential of recycling business in Lahore, Pakistan. Waste management, 28(2), 294-298. 6. 10.1016/j.wasman.2006.12.007 17475469

[15] Beaven RP. Hydrological and settlement behaviour of landfilled waste. Waste Manag. 2012;32(3):498–512.22188873

[16] Bhar P, Singh R, Sharma P. Methane emissions from waste: trends, sources, and mitigation approaches. Environ Sci Pollut Res Int. 2024;31(2):556–72.

[17] Christensen TH, Kjeldsen P, Bjerg PL, et al. Biogeochemistry of landfill leachate plumes: field and laboratory studies. Appl Geochem. 2001;16(7–8):659–718.

[18] El-Fadel M, Findikakis AN, Leckie JO. Environmental impacts of solid waste landfilling. J Environ Manage. 1997;50(1):1–25.

[19] El-Fadel, M., Bou-Zeid, E., Chahine, W., & Alayli, B. J. W. M. (2002). Temporal variation of leachate quality from pre-sorted and baled municipal solid waste with high organic and moisture content. Waste management, 22(3), 269-282. 2. 10.1016/s0956-053x(01)00040-x 11952174

[20] Hamid A, Asghar S. Determination of present household solid waste generation rate, physical composition and existing SWM practices in selected areas of Lahore. Nat Environ Pollut Technol. 2018;17(1):315–21.

[21] Hoornweg D, Bhada-Tata P. What a waste: a global review of solid waste management. World Bank; 2012.

[22] Hussain S, Khan Z, Malik RN, Riffat R. Assessment of leachate pollution index and its seasonal variation at an open dumpsite in Islamabad, Pakistan. Environ Monit Assess. 2020;192(9):569. 10.1007/s10661-020-08530-1.32770276

[23] Ibrahim TNT, Mahmood NZ, Othman FAR. Estimation of leachate generation from MSW landfills in Selangor. Environ Sci Technol. 2017;19(1):43–8.

[24] Ilmas B, Dongbei Y, Khalid S, Mir KA. Characterization and energy potential evaluation of urban municipal solid waste of Pakistan. Carbon Manag. 2021;12(6):581–91.

[25] Ilmas B, Mir KA, Khalid S. Greenhouse gas emissions from the waste sector: a case study of Rawalpindi in Pakistan. Carbon Manag. 2018;9(6):645–54.

[26] IPCC 2006b, Revised IEA. IPCC guidelines for national greenhouse gas inventories. In: Eggleston HS, Buendia L, Miwa K, Ngara T, Tanabe K (eds). IGES; Japan: p.10–11.

[27] Kawai K, Tasaki T. Revisiting estimates of municipal solid waste generation per capita and their reliability. J Mater Cycles Waste Manag. 2016;18(1):1–13.

[28] Kjeldsen P, Barlaz MA, Rooker AP, et al. Present and long-term composition of MSW landfill leachate: a review. Crit Rev Environ Sci Technol. 2002;32(4):297–336.

[29] Korai MS, Mahar RB, Uqaili MA. The feasibility of municipal solid waste for energy generation and its existing management practices in Pakistan. Renew Sustain Energy Rev. 2017;72:338–53.

[30] Kumar A, Samadder SR, Pandey V. A review on public health impacts of municipal solid waste landfills. Procedia Environ Sci. 2016;35:437–45. 10.1016/j.proenv.2016.07.049.

[31] Lakhouit A. Mitigating landfill emissions strategies for effective waste management in Tabuk. Cleaner Waste Systems. 2024;9:100187.

[32] U.S. EPA. Landfill Gas Emissions Model (LandGEM) Version 3.02 User’s Guide. Office of Research and Development, U.S. Environmental Protection Agency. 2005

[33] Leckie JO, Findikakis AN, El-Fadel M. Modeling landfill hydrology and leachate generation: developments and challenges. Waste Manag Res. 2020;38(12):1251–64.31902310

[34] Lou Z, Nair J. The impact of intermediate covers on landfill gas and leachate generation. Waste Manag. 2009;29(5):1522–9.

[35] Majumdar D, Ray S, Chakraborty S, Rao PS, Akolkar AB, Chowdhury M, et al. Emission, speciation, and evaluation of impacts of non-methane volatile organic compounds from open dump site. J Air Waste Manag Assoc. 2014;64(7):834–45. 25122957 10.1080/10962247.2013.873747

[36] Mir KA, Purohit P, Mehmood S. Sectoral assessment of greenhouse gas emissions in Pakistan. Environ Sci Pollut Res Int. 2017;24:27345–55. 28975514 10.1007/s11356-017-0354-y

[37] Choden Y, Pelzang K, Basnet AD, Dahal KB. Modeling of leachate generation from landfill sites. Nature Environment & Pollution Technology. 2022 1;21(3).

[38] Mor S, Kaur K, Ravindra K. Methane emissions from municipal landfills: a case study of Chandigarh and economic evaluation for waste-to-energy generation in India. Front Sustain Cities. 2024;6:1432995. 10.3389/frsc.2024.1432995.

[39] Munir S, Baqar M, Saeed N, Zameer M, Shaikh IA. Modeling greenhouse gases emissions from MSW of Lahore. Tech J UET Taxila. 2015;20(1):50.

[40] Pakistan Bureau of Statistics (PBS). 6th housing and population census 2017 report. Govt. of Pakistan 2017.

[41] Parvin F, Rahman MM, Rahman MA, Tareq SM. Assessment of leachate contamination and seasonal variation in chemical composition at Amin Bazar landfill site, Dhaka, Bangladesh. Environ Nanotechnol Monit Manage. 2021;15:100430. 10.1016/j.enmm.2021.100430.

[42] Qian X, Koerner RM, Gray DH. Geotechnical aspects of landfill design and construction. Prentice Hall; 2002.

[43] Ram C, Kumar A, Rani P. Municipal solid waste management: a review of waste to energy (WtE) approaches. Bioresour. 2021;16(2):4275.

[44] Renou S, Givaudan JG, Poulain S, Dirassouyan F, Moulin P. Landfill leachate treatment: review and opportunity. J Hazard Mater. 2008;150(3):468–93. 17997033 10.1016/j.jhazmat.2007.09.077

[45] Sharholy M, Ahmad K, Mahmood G, Trivedi RC. Municipal solid waste management in Indian cities – a review. Waste Manag. 2008;28(2):459–67. 10.1016/j.wasman.2007.02.008. 17433664 10.1016/j.wasman.2007.02.008

[46] Sharma A, Shenvi VV, Sain M. Reevaluating the concern of climate change. Int J Environ Clim Change. 2024;14:450–64.

[47] Sil D, Kumar P, Kumar D. Methane generation potential of municipal solid waste collected from different cities of India. Int J Environ Sci. 2014;4(5):822–30.

[48] Slack RJ, Gronow JR, Voulvoulis N. Household hazardous waste in municipal landfills: contaminants in leachate. Sci Total Environ. 2005;337(1–3):119–37. 10.1016/j.scitotenv.2004.07.002. 15626384 10.1016/j.scitotenv.2004.07.002

[49] Sohoo I, Qureshi S, Niazi M. Estimation of methane emissions from municipal solid waste disposal sites in Pakistan using the IPCC and LandGEM models. Sustain Environ Res. 2021;31(1):26. 10.1186/s42834-021-00099-7.

[50] Gupta J, Ghosh P, Kumari M, Thakur IS. Solid waste landfill sites for the mitigation of greenhouse gases. In: Biomass, biofuels, biochemicals 2022 (pp. 315-340). Elsevier

[51] Spokas KA, Bogner J, Corcoran M. Modeling landfill CH_4_ emissions: CALMIM international field validation, using CALMIM to simulate management strategies, current and future climate scenarios. Elem Sci Anth. 2021;9(1):00050.

[52] Talyan V, Dahiya RP, Sreekrishnan TR. State of municipal solid waste management in Delhi, the capital of India. Waste Manag. 2007;28(7):1276–87. 10.1016/j.wasman.2007.03.034. 17692510 10.1016/j.wasman.2007.05.017

[53] Thompson S, Sawyer J, Bonam R, Valdivia JE. Building a better methane generation model: validating models with methane recovery rates from 35 Canadian landfills. Waste Manag. 2009;29(7):2085–91. 10.1016/j.wasman.2009.02.032. 19328672 10.1016/j.wasman.2009.02.004

[54] Wakadikar A, Patil P, Joshi P. Biodegradation potential of different substrates by anaerobic microbial consortium. Int J Environ Sci. 2012;2(3):1270–8.

[55] Wang, J., & Qiao, Z. (2024). A comprehensive review of landfill leachate treatment technologies. Frontiers in Environmental Science, 12, 1439128. 4.

[56] Wijewardhana Y, Dissanayake D, Gunawardena S. Seasonal variation of landfill leachate and its impact on groundwater quality: a case study from Colombo, Sri Lanka. Environ Chall. 2022;7:100474. 10.1016/j.envc.2022.100474.

[57] Williams, P. A., Narra, S., Antwi, E., Quaye, W., Hagan, E., Asare, R., ... & Ekanthalu, V. S. (2023, March). Review of barriers to effective implementation of waste and energy management policies in Ghana: Implications for the promotion of waste-to-energy technologies. In Waste (Vol. 1, No. 2, pp. 313- 332). MDPI. 3.

[58] Yaashikaa PR, Kumar PS, Nhung TC, Hemavathy RV, Jawahar MJ, Neshaanthini JP, et al. A review on landfill system for municipal solid wastes: insight into leachate, gas emissions, environmental and economic analysis. Chemosphere. 2022;309:136627. 36181852 10.1016/j.chemosphere.2022.136627

[59] Zhang Y, Li Q, Wang H. Application of hydrological models (HELP, HYDRUS) in leachate prediction under varying cover systems. Environ Earth Sci. 2021;80(17):563.

[60] Zuberi MJS, Ali SF. Greenhouse effect reduction by recovering energy from waste landfills in Pakistan. Renew Sustain Energy Rev. 2015;44:117–31.

[61] Zuberi MJS, Torkmahalleh MA, Ali SH. A comparative study of biomass resources utilization for power generation and transportation in Pakistan. Int J Hydrogen Energy. 2015;40(34):11154–60.

[62] Asian Development Bank ADB. Pakistan National Urban Assessment. 2024 Available from 10.22617/tcs240377-2

[63] IPCC 2006a. 2006 IPCC guidelines for national greenhouse gas inventories: volume 5 – waste. Intergovernmental Panel on Climate Change. 2006. Retrieved from https://www.ipcc-nggip.iges.or.jp/public/2006gl/vol5.html

[64] Pakistan Environmental Protection Agency (Pak-EPA). Hospital and municipal waste in Pakistan: current management practices and guidelines. ministry of environment, government of Pakistan. 2005

[65] UNEP. Waste Management Outlook for Asia and the Pacific. United Nations Environment Programme. 2019

[66] United Nations Environment Programme, UNEP. Waste Management Outlook for Asia and the Pacific. United Nations Publications; 2016.

[67] US EPA. Overview of greenhouse gases: Methane emissions. United States Environmental Protection Agency. 2013. https://www.epa.gov/ghgemissions/overview-greenhouse-gases#methane

[68] World Health Organization (WHO). Ambient air pollution: A global assessment of exposure and burden of disease. 2016. https://www.who.int/publications/i/item/9789241511353

